# Regulation of Oxidative Stress in Corneal Endothelial Cells by Prdx6

**DOI:** 10.3390/antiox7120180

**Published:** 2018-12-04

**Authors:** Matthew Lovatt, Khadijah Adnan, Gary S. L. Peh, Jodhbir S. Mehta

**Affiliations:** 1 Tissue Engineering and Stem Cell group, Singapore Eye Research Institute (SERI), Singapore 169856, Singapore; khadijah.adnan@seri.com.sg (K.A.); garypeh@gmail.com (G.S.L.P.); 2 Eye-ACP, Duke-NUS Graduate Medical School, Singapore 169857, Singapore; 3 Singapore National Eye Centre (SNEC), Singapore168751, Singapore; 4 School of Material Science and Engineering, Nanyang Technological University, Singapore 639977, Singapore

**Keywords:** Prdx6, cornea, Fuchs’ endothelial corneal dystrophy, lipid peroxidation, mitochondrial membrane potential

## Abstract

The inner layer of the cornea, the corneal endothelium, is post-mitotic and unable to regenerate if damaged. The corneal endothelium is one of the most transplanted tissues in the body. Fuchs’ endothelial corneal dystrophy (FECD) is the leading indication for corneal endothelial transplantation. FECD is thought to be an age-dependent disorder, with a major component related to oxidative stress. Prdx6 is an antioxidant with particular affinity for repairing peroxidised cell membranes. To address the role of Prdx6 in corneal endothelial cells, we used a combination of biochemical and functional studies. Our data reveal that Prdx6 is expressed at unusually high levels at the plasma membrane of corneal endothelial cells. RNAi-mediated knockdown of Prdx6 revealed a role for Prdx6 in lipid peroxidation. Furthermore, following induction of oxidative stress with menadione, Prdx6-deficient cells had defective mitochondrial membrane potential and were more sensitive to cell death. These data reveal that Prdx6 is compartmentalised in corneal endothelial cells and has multiple functions to preserve cellular integrity.

## 1. Introduction

The cornea is a transparent refractive structure at the front of the eye. The cornea is approximately 500 μm thick and composed of several layers, including a protective outer epithelial cell layer; an acellular Bowman’s layer; a stromal layer; an acellular Descemet’s membrane; and a single uniform layer of cells on the posterior surface, termed the corneal endothelium (CE) [1,2,3]. Corneal endothelial cells (CEnCs) are neural crest derived and have striking hexagonal morphology. The CE is essential for maintaining corneal hydration and therefore optimal corneal transparency. This is achieved through a pump-leak mechanism: nutrients from the aqueous humor are allowed to passively diffuse through the ‘leaky’ endothelium into the stroma and, concurrently, an ionic pump drives fluid from the stroma into the aqueous humor. This dynamic ‘barrier and pump’ function serves to maintain corneal hydration and transparency vital for visual acuity.

Corneal endothelial cells are growth-arrested and cannot regenerate [1,2,3]. Age-dependent degeneration and decline in number of CEnCs is known to occur [4,5]. Furthermore, CEnCs are metabolically active and subjected to persistent UV exposure. Indeed, UV exposure is known to induce DNA damage and apoptosis of CEnCs [6]. Therefore, to maintain functional integrity and corneal transparency, CEnCs must possess intrinsic mechanisms to control their level of oxidative stress. Damage to the CE due to age-related degeneration or genetic corneal dystrophies is one of the leading causes of corneal blindness. Currently, CEnC dysfunction can only be remedied by a corneal transplant, which replaces the dysfunctional cell layer and restores vision. Corneal tissue is the most transplanted tissue in the body.

The most common corneal endothelial dystrophy is Fuchs’ endothelial corneal dystrophy (FECD) [7]. A number of factors, including age, sex, and several genetic loci, are implicated in FECD. However, oxidative stress is also thought to contribute to the pathogenesis of FECD: cornea tissues from Fuchs’ patients display an overall increase in reactive oxygen species (ROS), and human CEnC cell lines derived from FECD patients are more vulnerable to oxidative insults [8]. Interestingly, proteomic analysis of pooled CE from FECD patients has revealed a specific downregulation of the peroxiredoxin (Prdx) family of antioxidants [9].

The Prdx protein family contains six isoforms that are capable of reducing hydrogen peroxide and alkyl hydroperoxides. The Prdx family is characterised by a conserved catalytic cysteine (Cys) in the N-terminus. All Prdx proteins, with the exception of Prdx6, additionally contain an additional conserved resolving cysteine in the C-terminus. However, Prdx6 possesses only a single *N*-terminus catalytic cysteine. Furthermore, Prdx6 is the only member of the family to possess both phospholipase A2 (PLA_2_) activity and lysophosphatidylcholine acyl transferase (LPCAT) activity [2,10,11]. Peroxides such as hydrogen peroxide oxidize the N-terminal Cys to form sulfenic acid. Normally, an intermolecular disulfide bond is formed with a second adjacent C-terminal Cys residue, which is then reduced by thioredoxin to complete the catalytic cycle. However, for 1-Cys Prdx6, glutathione is used as the reductant [12].

Although Prdx6 is primarily a cytosolic protein, its recruitment to the plasma membrane is thought to be essential for its role in repairing peroxidised membranes [13]. We recently identified an antibody, TAG-2A12, as a unique marker for CEnCs [14]. Interestingly, immunohistochemistry and flow cytometry revealed that TAG-2A12 labelled the cell surface of CEnC. The target antigen bound by TAG-2A12 was identified to be Prdx6 by mass spectrometry [14]. However, by flow cytometry, commercially available antibodies to Prdx6 do not label the cell surface of CEnCs, suggesting that TAG-2A12 recognises an epitope associated with membrane-bound Prdx6. This was the first report of the expression of this protein on the corneal endothelial surface. However, membrane Prdx6 has also been reported in activated neutrophils [15] as well as in the human lung carcinoma cell line, A549, treated with peroxide [16]. It has been postulated that cytosolic Prdx6 can translocate to the plasma membrane, bind oxidised phospholipids, and perform a fundamental role in repair of peroxidised cell membranes [13].

In this study, we aimed to confirm the cell surface expression of Prdx6 and to characterise the function of Prdx6 in human CEnCs. We report that high levels of Prdx6 can be isolated from the plasma membrane of CEnCs. The level of Prdx6 diminishes during oxidative stress. Using siRNA to target Prdx6 in a CE cell line, we demonstrate that Prdx6 is required to maintain both lipid peroxidation as well as cell viability through the regulation of mitochondrial function.

## 2. Material and Methods

### 2.1. Chemicals and Reagents

All reagents were purchased from Sigma (now under Merck, Darmstadt, Germany) unless otherwise stated.

### 2.2. Induction of Oxidative Stress

Specific reagents were selected to induce oxidative stress in different cellular compartments. Cumene hydroperoxide (CH), which is lipophilic, was selected because it has been previously demonstrated to induce lipid peroxidation at the plasma membrane [17]. Tert-butyl hydroperoxide (tBHP) was selected as a stable alternative to hydrogen peroxide that initiates reactive oxygen species (ROS) and induces lipid peroxidation and apoptosis. Menadione (MN) was used to increase intracellular ROS. Furthermore, MN treatment of normal CEnCs has been demonstrated to mimic pathological changes seen in ex vivo FECD specimens [18]. Suitable doses of each compound were chosen based on either manufacturers’ instructions or published data, or were empirically determined.

### 2.3. Human Corneal Tissue and Cell Culture

Primary human corneal endothelial cells (pCEnCs) obtained from cadaveric corneal tissue unsuitable for transplantation were procured through Lions Eye Institute for Transplant and Research (Tampa, FL, USA). Isolated pCEnCs were expanded using a dual-media approach until passage 2 or passage 3, in 6 cm plates, as previously described [19]. Thereafter, cultures were maintained in M5 media consisting of Human endothelial-SFM (Life Technologies, Thermo Fisher Scientific, Waltham, MA, USA) supplemented with 5% serum (EquaFetal^®^ Atlas Biologicals, Fort Collins, CO, USA). To isolate corneal stromal fibroblasts, stromal tissue fragments were digested with collagenase I (1 mg/mL, Worthington, Lakewood, NJ, USA) for 24 h at 37 °C. Single cells were suspended in Dulbecco’s Modified Eagle Medium (DMEM) 10% bovine serum and cultured until confluent. Human retinal microvascular endothelial cells (RMECs, Angio Proteomie, cAP-0010) were maintained in M5 10% serum and used at passage 5.

The SV40-transformed human corneal endothelial cell line (B4G12, [20]), the human lung adenocarcinoma A549 cell line (ATCC; CCL-185), HEK293T, and the spontaneous arising retinal pigmented epithelial cell line ARPE19 were maintained at 37 °C, 5% CO_2_ in DMEM (high glucose) supplemented with 10% bovine serum (Gibco; Thermo Fisher Scientific, Waltham, MA, USA) together with antibiotics.

Where indicated, cells were treated with tert-butyl hydroperoxide (tBHP) 500 μm for 3 h at 37 °C.

### 2.4. Cell Fractionation and Western Blotting

Cell surface proteins were isolated using the Pierce Cell Surface Isolation Kit (Thermo Fisher Scientific). Briefly, confluent cultures of cells were incubated with the cell membrane impermeable, cleavable biotinylation reagent EZ-Link™ Sulfo-NHS-SS-Biotin at 4 °C for 30 min. Following cell lysis, labelled proteins were isolated by incubation with NeutrAvidin agarose for 1 h. Following extensive washing, proteins were eluted in sodium dodecyl sulfate-polyacrylamide gel electrophoresis (SDS-PAGE) sample buffer containing 50 mM dithiothreitol (DTT).

#### Plasma Membrane Isolation

Plasma membrane fractions were isolated using the Plasma Membrane Protein Extraction Kit (Abcam, ab65400, Cambridge, UK). Briefly, cells were chilled on ice, washed in ice-cold phosphate buffered saline (PBS), and scraped into ice-cold PBS. Cell pellets were lysed in homogenization buffer containing protease inhibitors. Homogenization was completed with low power sonication at 4 °C. Total membrane proteins were pelleted by centrifugation at 10,000× *g* for 30 min at 4 °C. The supernatant (cytoplasmic fraction) was removed. Plasma membrane proteins were purified by resuspending the total membrane pellet in a combination of lower phase/upper phase solutions, and centrifugation. The exact constituents of these solutions is proprietary, but most likely based on an aqueous polymer two-phase separation system which separates plasma membranes based on their affinity for two immiscible polymers, such as, polyethylene glycol and dextran [21]. Membrane pellets were dissolved in 0.5% Triton X-100 in PBS. Proteins were quantitated by BCA assay (Pierce, Thermo Fisher Scientific) and equivalent amounts loaded on 4–20% mini-PROTEAN^®^ TGX™ Gels (Bio-Rad, Hercules, CA, USA). Gels were transferred to PVDF membranes and blocked in 5% non-fat milk. The following antibodies were used for immunoblotting: PRDX6 (4A3, ab16947, Abcam), CD325 (N-Cadherin, clone 8C11,), and β-Catenin (clone 14) (both from BD Biosciences, San Jose, CA, USA). Na^+^/K^+^-ATPase (sc71638, Santa Cruz Biotechnology, Dallas, TX, USA) and GAPDH (clone FF26A/F9 and β-Actin clone 2F1-1, both BioLegend) served as loading controls. Blots were washed in PBST (PBS + 0.1% tween-20), probed with HRP-conjugated secondary antibodies (Cell signalling Technology, Danvers, MA, USA), and visualised by chemiluminescence. Bands were quantified using ChemiDoc™ MP imaging system and image lab software (Bio-Rad, Hercules, CA, USA).

### 2.5. RNAi Knockdown of Prdx6

Confluent cultures of B4G12 cells were harvested and seeded in 12-well plates at 40k/cm^2^. Cells were transfected with 10 μm Silencer^®^ select validated siRNA (Ambion^®^ by Life Technologies, Thermo Fisher Scientific, Waltham, MA, USA) together with Lipofectamine™ RNAiMAX transfection reagent (Thermo Fisher Scientific) at the time of seeding, according to the manufacturers’ instructions. The following siRNA reagents were used: Silencer^®^ select Prdx6 (ID# s18430) and, as control, Silencer^®^ select negative control #1. After 24 h of culture, media was changed and cells were re-transfected. Cells were analysed the following day. Knockdown of Prdx6 was confirmed by 48 h post-transfection by directly lysing cells in SDS-PAGE sample buffer and probing western blots with anti-Prdx6 antibodies. Bands were quantified using ChemiDoc™ MP imaging system and image lab software (Bio-Rad, Hercules, CA, USA). Alternatively, knockdown of Prdx6 was analysed by real-time PCR analysis. Briefly, total RNA was extracted using RNeasy kit (Qiagen, Venlo, Netherlands) and 500 ng was reversed transcribed with iSCRIPT (Bio-Rad). Real-time PCR was performed using TaqMan^®^ gene expression assays (Thermo Fisher Scientific). Relative quantification was normalised using GAPDH and calculated by 2^−ΔΔ*C*t^.

### 2.6. Lipid Peroxidation Assay

Lipid peroxidation was measured using the Click-iT™ Lipid Peroxidation Imaging Kit (Thermo Fisher Scientific) according to the manufacturer’s instructions, with modifications for adaptation for flow cytometry. Briefly, B4G12 cells were treated with 100 μm CH in media containing 50 μm linoleamide alkyne (LAA) for 2 h at 37 °C. Cells were harvested by cell dissociation in TrypLE Express (Thermo Fisher Scientific) and washed in PBS/0.1% bovine serum albumin (BSA). Cells were pelleted by centrifugation and resuspended in 100 μL of 3.7% paraformaldehyde (PFA) for 15 min at room temperature. Cells were washed in PBS/0.1% BSA and resuspended in PBS/0.05% Triton X-100 for 10 min at room temperature. Next, 1 mL PBS/1% BSA was directly added to cells and incubated for 20 min at room temperature. Cells were pelleted and gently washed in PBS without BSA. Cell pellets were incubated in Click-iT reaction cocktail containing Alexa Fluor 488 azide in the presence of CuSO_4_, for 30 min at room temperature protected from light. Cells were immediately analysed on a BD FACSVerse™ flow cytometer (BD Biosciences).

### 2.7. Cell Viability Assay: Flow Cytometry

Transfected B4G12 cells were treated with CH (100 μm) for 4 h at 37 °C in a tissue culture incubator. Cells were washed in PBS and harvested by trypsinisation. Aliquots (100 μL) of cells were transferred to Eppendorf tubes and incubated with 5 μL of FITC-Annexin V and 10 μL of PI (BioLegend) for 15 min at room temperature in Annexin V binding buffer. Cells were re-suspended in 400 μL Annexin V buffer and immediately analysed on a BD FACSVerse™ flow cytometer.

#### 2.7.1. Cell Viability Assay: xCELLigence

Cell viability was assessed using the xCELLigence real-time cell analyser (ACEA Bioscience, Agilent, San Diego, CA, USA). siRNA-transfected B4G12 cells were seeded at 4 × 10^4^ in triplicate in an E-Plate 96 (ACEA Biosciences) and left to attach for 18 hrs. Cells were left untreated or treated with indicated compounds. The electrical impedance readings of overall cell viability were recorded, using the xCELLigence real-time cell analyser, throughout the experiment for at least 8 h. The percentage of viable cells was calculated for each time point relative to time zero, which was set as the last impedance reading prior to addition of compounds. Data shown are representative of three independent experiments. Statistical analysis was performed using GraphPad software (Prism, version 7, GraphPad, San Diego, CA, USA). Groups were compared with two-way ANOVA, followed by post-hoc Bonferroni test, for multiple comparisons, with *p*-value < 0.05 considered significant.

#### 2.7.2. Cell Viability Assay: Mitochondrial Membrane Potential (ΔΨm)

Mitochondrial membrane potential was measured using the TMRE assay (Cell Signalling Technology). Briefly, siRNA-transfected cells were treated with 50 μm MN for 90 min or the potent ΔΨm disruptor CCCP (50 μm) for 15 min. The mitochondrial membrane dye, TMRE, was added at a final concentration of 200 nM for 20 min. Cells were trypsinised and immediately analysed by flow cytometry.

## 3. Results

### 3.1. Membrane Expression of Prdx6 in Corneal Endothelial Cells

**Figure 1:**
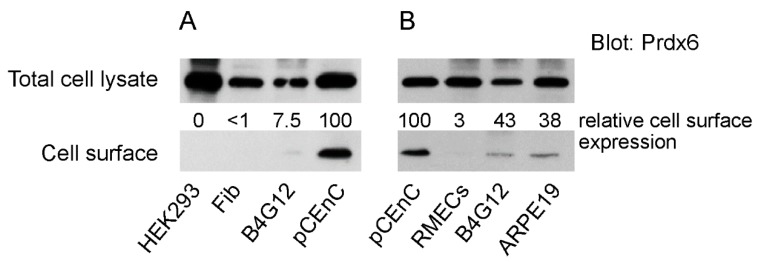
(**A**,**B**) Prdx6 is predominantly expressed at the cell surface in human corneal endothelial cells (CEnCs). The indicated cells had their cell surface biotinylated using Sulfo-NHS-SS-Biotin. Cell lysates were subjected to immunoprecipitation with NeutrAvidin™ agarose. Eluted proteins were probed with Prdx6 antibodies. For comparative purposes, an aliquot of cell lysate prior to immunoprecipitation was used for analysis of total cellular Prdx6 levels. Bands were quantitated, and relative surface Prdx6 levels were normalised against total Prdx6 levels. Primary human corneal endothelial cell (pCEnC) levels were arbitrarily set to 100. Fib: corneal fibroblasts. RMECs: retinal microvascular endothelial cells.

We compared the membrane localisation of Prdx6 in primary and transformed cell lines derived from the eye. We incorporated in our analysis the human CEnC line B4G12 [20], as well as the spontaneous arising retinal epithelial cell line ARPE19. As a non-eye control, we used human embryonic kidney cells (HEK293T). Cell surface proteins were isolated by biotinylation of cells and subsequent immunoprecipitation with neutravidin agarose, as detailed in Section 2.4. Western blotting of eluted surface proteins with Prdx6 antibodies revealed that Prdx6 was primarily detected in pCEnCs (Figure 1A). Prdx6 was similarly detected in B4G12 cells, albeit at low levels. No detectable surface Prdx6 was detected in HEK293T or corneal stromal fibroblasts, despite the abundance of cytosolic Prdx6 (Figure 1A). To verify that Prdx6 can be detected at the cell surface in B4G12 cells, we repeated our experiments using a greater number of B4G12 cells, as well as pCEnCs isolated from fresh donors (Figure 1B). Corroborating our previous result, high surface Prdx6 levels were detected in pCEnCs (Figure 1B). Low levels of Prdx6 were also detected in B4G12 cells. The surface level of Prdx6 was not unique to pCEnCs because the cell line ARPE19 similarly had detectable expression of Prdx6 in cell surface extracts. However, the greatest surface levels of Prdx6 were detected in pCEnCs.

### 3.2. Prdx6 Levels are Sensitive to Oxidative Stress

**Figure 2:**
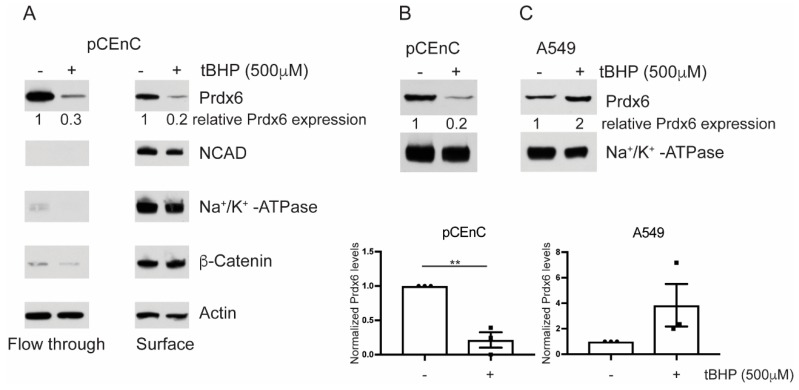
Prdx6 levels are sensitive to oxidative stress. (**A**) Primary cultures of human CEnCs) were treated with tert-butyl hydroperoxide (tBHP) for 3 h. pCEnCs were biotinylated and cell surface proteins purified by immunoprecipitation. The flow-through, unbound fraction served as a control for membrane fractionation. (**B**) pCEnCs and (**C**) A549 cells were treated with tBHP for 3 h and plasma membrane was isolated by density dependent centrifugation. Data from three independent experiments ± SEM is shown. Prdx6 levels were normalized to Na^+^/K^+^-ATPase and expressed relative to untreated (−) controls. Student *t*-test was performed to evaluate statistical significance (** *p*-value < 0.005).

Western blot analysis of the cell surface fraction, from pCEnCs treated with tert-butyl hydroperoxide (tBHP), revealed that Prdx6 levels were diminished in pCEnCs under oxidative stress. The affect was apparent for both cell surface-bound Prdx6 as well the unbound fraction. Importantly, membrane-levels of N-Cadherin, β-Catenin, and Na^+^K^+^-ATPase were not affected by tBHP treatment (Figure 2A). Membrane proteins, such as N-Cadherin and Na^+^K^+^-ATPase, were not detected in the unbound flow-through fraction, which validated our cell surface isolation protocol. To confirm these data, we employed an alternative protocol for the isolation of plasma membrane (PM) proteins (Section 2.4). Purified PM proteins were extracted from untreated or tBHP treated pCEnCs. Similar to our previous observation, Prdx6 levels were downregulated during oxidative stress (Figure 2B). As controls for these experiments, we used the lung carcinoma A549 cells. In contrast to pCEnCs, A549 demonstrated an overall increase in PM bound Prdx6 (Figure 2B and Figure S1), although statistical analysis revealed the differences to be insignificant. However, our data is consistent with previous reports demonstrating translocation of Prdx6 to the PM following tBHP treatment [16]. In B4G12 cells, plasma membrane Prdx6 was not altered by tBHP, despite a decrease in levels of cytoplasmic Prdx6 following tBHP treatment (Figure S1). These results suggest subtle differences between primary and transformed CEnCs.

Taken together, our data suggest that CEnCs have Prdx6 constitutively associated with the plasma membrane.

### 3.3. Prdx6 is Required for a Normal Lipid Peroxidation Response

**Figure 3:**
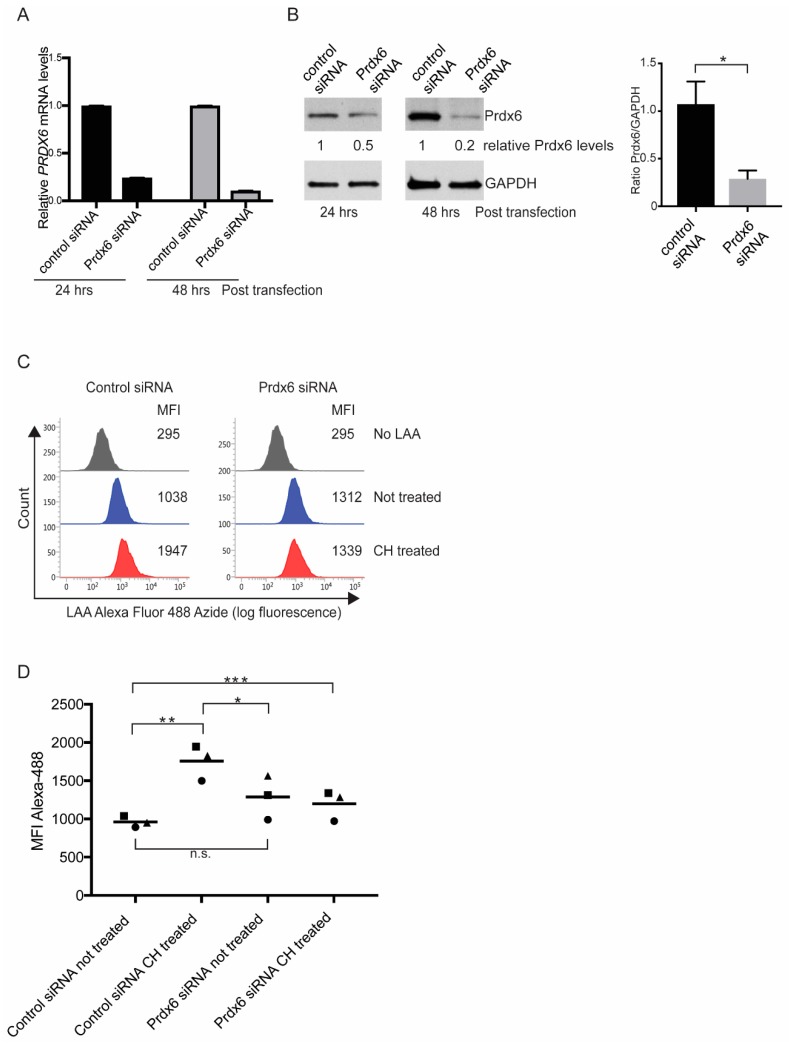
Targeted knockdown of Prdx6 in CEnCs reveals a role for Prdx6 in regulating lipid peroxidation. (**A**) Prdx6 mRNA levels were determined by qPCR analysis. Expression levels are shown relative to control siRNA treated B4G12 CEnCs and normalized to GAPDH. (**B**) Representative western blot analysis of aliquots of siRNA-transfected B4G12 CEnCs probed with anti-Prdx6 antibodies. Protein levels were quantitated relative to GAPDH and normalised to control siRNA. Data also expressed as mean ± SEM (*n* = 6). * *p*-value (<0.05) was determined by student *t*-test. (**C**) Lipid peroxidation was measured in B4G12 CEnCs incubated with linoleamide alkyne (LAA) in the presence (cumene hydroperoxide (CH) treated) or absence of CH (not treated). B4G12 CEnCs incubated in the absence of LAA served as negative controls. (**D**) Data are expressed as mean fluorescence intensity (MFI) from three independent experiments. Two-way ANOVA with multiple comparisons was used to test statistical significance. Asterix (*) indicates statistical significance; * *p* < 0.05, ** *p* < 0.005, *** *p* < 0.01, n.s.: no significant difference.

To elucidate the function of Prdx6 in CEnCs, we employed RNAi-mediated knockdown of Prdx6 in CEnCs. Our initial attempts to knockdown Prdx6 in pCEnCs did not reveal significant loss of gene and/or protein expression. Therefore, we utilised the CE cell line B4G12. B4G12 cells were amenable to transient transfection of siRNA, resulting in downregulation of Prdx6 mRNA at 24 and 48 h post-transfection (Figure 3A). This resulted in a ~80% knockdown efficiency of protein 48 h post-transfection compared to B4G12 cells transfected with negative control siRNA reagents (Figure 3B).

To explore the influence of Prdx6 on cellular membranes, we treated B4G12 cells with cumene hydroperoxide (CH) and measured lipid peroxidation by flow cytometry. In cells transfected with control siRNA, CH induced lipid peroxidation, as judged by a ~2-fold increase in mean fluorescent (MFI) intensity of the Alexa Fluor 488 fluorophore (Figure 3C,D). Interestingly, the level of lipid peroxidation in untreated Prdx6 knockdown B4G12 cells was slightly higher compared to controls. However, this was not statistically significant (Figure 3D). Surprisingly, in response to CH, B4G12 cells lacking Prdx6 were unable to respond to CH and the fluorescence intensity of LAA-AF remained comparable to untreated cells (Figure 3C,D).

### 3.4. Loss of Prdx6 Does Not Affect Cell Viability in Response to Cumene Hydroperoxide

**Figure 4:**
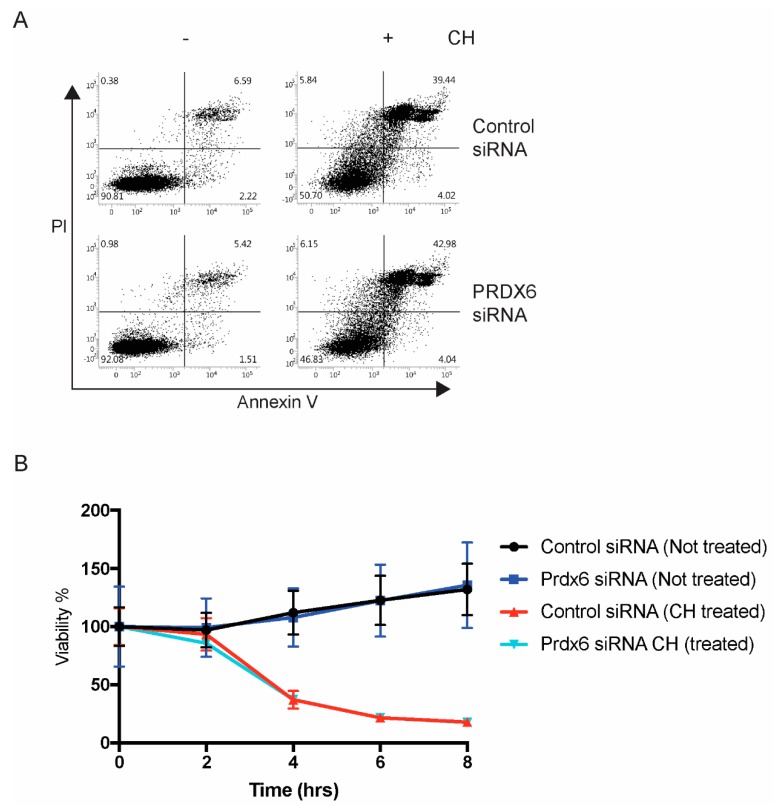
Normal apoptosis in the absence of Prdx6. (**A**) B4G12 CEnCs were treated with CH (100 μm) for 4 h and stained with Annexin V and propidium iodide (PI). Representative FACS plots are shown and the percentages of viable (AnV^−^PI^−^), early, (AnV^+^PI^−^), late apoptotic (AnV^+^PI^+^), and necrotic (AnV^−^PI^+^) are shown. (**B**) B4G12 CEnC cells were seeded on xCELLigence plates and continuously monitored for 8 h after the addition of CH (50 μm).

To explore whether loss of Prdx6 will affect apoptosis, we labelled B4G12 cells with Annexin V and propidium iodide (PI) following exposure to CH for 4 h. In response to CH, a large proportion (~40%) of cells were judged to be apoptotic (AnV^+^/PI^+^) in both control and Prdx6 siRNA-transfected B4G12 cells. However, the response between control and Prdx6-deficient cells to CH was not statistically significant (Figure 4A). To verify these data, we employed xCELLigence for real-time monitoring of cell viability. The addition of CH to both control and Prdx6-deficient cells resulted in a time-dependent decrease in cell viability with overlapping kinetics (Figure 4B), suggesting Prdx6 expression is not required to inhibit apoptosis in CEnCs.

### 3.5. Regulation of Mitochondrial Membrane Potential by Prdx6

**Figure 5:**
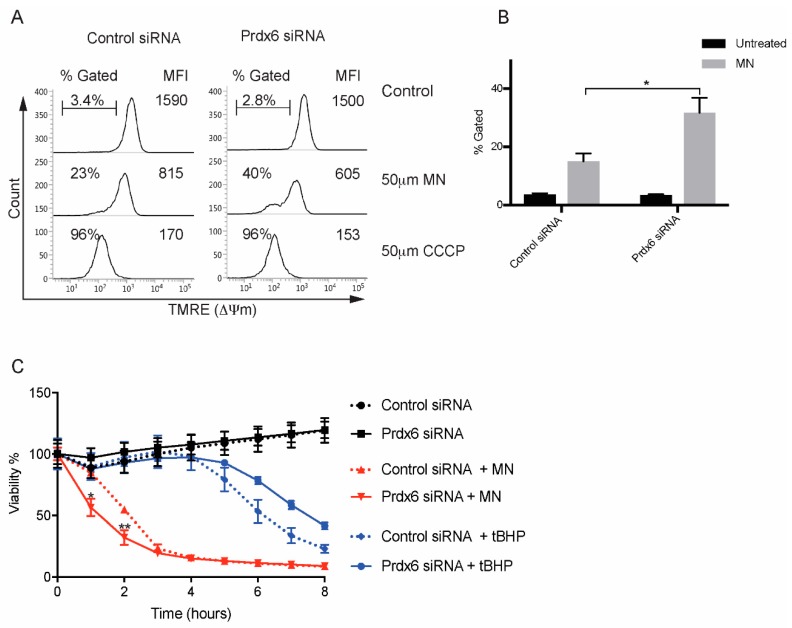
Prdx6-deficient cells are susceptible to cell death induced by menadione MN. (**A**) Representative FACS plots of TMRE mitochondrial membrane potential (ΔΨm) in siRNA-transfected B4G12 cells following treatment with 50 μm MN (90 min) or 50 μm CCCP (15 min). Percentage of cells with loss of ΔΨm is shown as well as overall TMRE mean fluorescence intensity (MFI). (**B**) Data are presented as mean values ± SEM (*n* = 4) of percentage TMRE negative. Two-way ANOVA reveals statistical significance (*p < 0.005) between MN treated control and Prdx6 siRNA-transfected cells. (**C**) Viability in response to MN and tBHP was assessed as in Figure 4B. Data is presented as mean values ± SEM (*n* = 3). Statistical analysis for each time point was carried out using two-way ANOVA with Bonferroni post-hoc multiple comparisons test (* *p* < 0.0001, ** *p* < 0.001).

Treatment of CEnCs with menadione (MN) has been demonstrated to reduce mitochondrial membrane potential (ΔΨm), induce mitochondrial dysfunction, and to partially mimic phenotypes associated with FECD [18]. Preliminary data demonstrated that the potent ΔΨm disruptor CCCP caused a significant loss in TMRE fluorescence, indicating loss of ΔΨm in both control and Prdx6 siRNA-transfected B4G12 cells. However, MN did not cause ΔΨm in this time frame (Figure 5A and Figure S2). Therefore, we performed time courses to establish whether MN induces ΔΨm with prolonged incubation (Figure S2). Compared to tBHP, MN induced a more robust ΔΨm in a time-dependent fashion (Figure S2). Following a 90-min exposure to MN, differences between control and Prdx6 knockdown cells could be detected, with Prdx6 knockdown cells displaying greater loss of ΔΨm compared to controls (Figure 5A). Therefore, in response to MN, loss of Prdx6 influences mitochondrial function.

We treated control and Prdx6 knockdown B4G12 cells with MN or tBHP and measured cellular viability with xCELLigence (Figure 5B). Compared to tBHP, MN rapidly decreased cell viability in control and Prdx6 knockdown B4G12. However, for Prdx6-deficient cells, loss of viability was consistently accelerated compared to controls. The augmented cell death in Prdx6 knockdown cells was not seen with tBHP, suggesting the effects are specific to MN (Figure 5B). Taken together, it appears that loss of Prdx6 renders B4G12 cells more susceptible to oxidative stress and accelerates cell death in response to MN.

## 4. Discussion

Corneal endothelial cells are susceptible to oxidative stress. In part, FECD is thought to be an oxidative stress disease [22]. The late onset of FECD suggests that the continual exposure to UV-induced oxidative stress gradually renders endothelial cells in FECD more susceptible to oxidative damage. Consistent with this, a decrease in Prdx (including Prdx6) levels has been demonstrated [9]. Furthermore, an increase in oxidative damage in ex vivo FECD tissue has been reported [8]. Menadione induces intracellular ROS in cultures of normal human CEnCs and produces effects similar to that seen in cells derived from FECD tissue [18].

We report that Prdx6 levels are compartmentalised to the plasma membrane in both primary cultures of CEnCs as well as the CE cell line B4G12. In A549 cells Prdx6 translocates to the plasma membrane in response to oxidative stress in order to protect the cell membrane. We reasoned that Prdx6 might play a similar function in CEnC. However, in primary CEnCs oxidative stress resulted in a decrease in both cytosolic as well as PM Prdx6 levels. Likewise, cytoplasmic Prdx6 in B4G12 was reduced following oxidative stress. However, the PM levels did not change within the 3-h timeframe. In an attempt to elucidate the function of plasma membrane associated Prdx6, we performed knockdown of Prdx6 followed by treatment with CH. Cumene hydroperoxide induces lipid peroxidation at the plasma membrane with no effect on mitochondria [17]. We predicted that Prdx6-deficient cells would have increased sensitivity to lipid peroxidation. However, in response to CH, the level of lipid peroxidation did not increase, suggesting Prdx6 is required for cells to respond to CH. Our data on Prdx6 knockdown of B4G12 endothelial cells contradicts that described for pulmonary microvascular endothelial cells (PMVECs) isolated from the lungs of Prdx6-deficient mice. In response to tBHP, Prdx6-deficient PMVECs are more sensitive to lipid peroxidation [13]. Interestingly, both peroxidase as well as PLA_2_ activity was required for Prdx6 to mediate membrane repair following lipid peroxidation [13]. The discrepancy between these results is unclear. Both CH and tBHP induce lipid peroxidation and apoptosis to the same extent. We can only speculate that in the absence of Prdx6, compensatory mechanisms might be induced to maintain cell membrane integrity in CEnC.

Alternatively, Prdx6 might actively contribute to lipid peroxidation. Indeed, following oxidative stress, Prdx6 PM levels were rapidly diminished, an effect not seen with other PM proteins. In this scenario, loss of Prdx6 may be protective. Indeed, viability in the absence of Prdx6 was greater compared to controls in response to tBHP. However, in response to CH, apoptosis was comparable between control and Prdx6 siRNA-transfected B4G12 cells (Figure 4A). This suggests that the level of Prdx6 may impact how cells respond to different types of ROS. These data do not rule out that non-apoptotic cell death pathways such as ferroptosis [23] might be affected by Prdx6. Indeed, similar to Prdx6, GPx4 also uses GSH as its physiological reductant, (which is required for repair of peroxidised membranes), and loss of its expression drives ferroptosis [24].

Prdx6 is known to translocate to the PM in activated neutrophils and associate with components of the NADPH oxidase complex, and is required to enhance the generation of superoxide [15]. Whether or not PM Prdx6 in pCEnCs is associated with components of the NADPH oxidase is not currently known. A role for Prdx6 in generating superoxide may appear contradictory to the antioxidant role of Prdx6. However, localised peroxide is emerging as a niche for signal transduction. For example, localised inactivation of Prdx1 in lipid rafts is required for the generation of highly localised H_2_O_2_ within a signalling microdomain [25]. Currently it remains unclear as to why CEnCs have high Prdx6 levels at the PM.

Prdx6 has been demonstrated to be rapidly recruited to mitochondrial in CCCP-treated HeLa cells and is required to block the induction of mitophagy [26]. Prdx6 has also been revealed to translocate to mitochondria in hepatocytes in response to an ischemia-reperfusion injury model [27]. Furthermore, Prdx6-deficient mice have increased generation of mitochondrial-produced H_2_O_2_ and increased hepatocyte injury in response to ischemia-reperfusion injury [27]. Interestingly, loss of mitochondrial membrane potential and induction of mitophagy is strongly implicated in FECD [18,28].

## 5. Conclusions

Consistent with these findings, we report that loss of Prdx6 results in heightened loss of mitochondrial membrane potential, together with accelerated cell death in B4G12 in response to MN. Taken together, our data suggest that oxidative stress results in a decrease in Prdx6. In turn, mitochondria become more sensitive to oxidative damage, which we suggest is responsible for the increased mitochondrial dysfunction apparent in FECD.

## Supplementary Materials

The following are available online at http://www.mdpi.com/2076-3921/7/12/180/s1, Figure S1. Cytoplasmic and plasma membrane (PM) proteins were purified from A549 and B4G12 cell lines left untreated (−) or treated (+) for 3 h with tBHP. Figure S2. Menadione disrupts mitochondrial membrane potential. B4G12 cells were treated with the indicated compounds for the desired times.
