# Supplementary material for: Regulation of Oxidative Stress in Corneal Endothelial Cells by Prdx6
Source: Antioxidants (Basel). 2018 Dec 4;7(12):180. doi: 10.3390/antiox7120180 (PMC6316742; doi:10.3390/antiox7120180)
Supplement: Supplementary file 1 [file antioxidants-07-00180-s001.pdf]

antioxidants

Supplementary Materials:

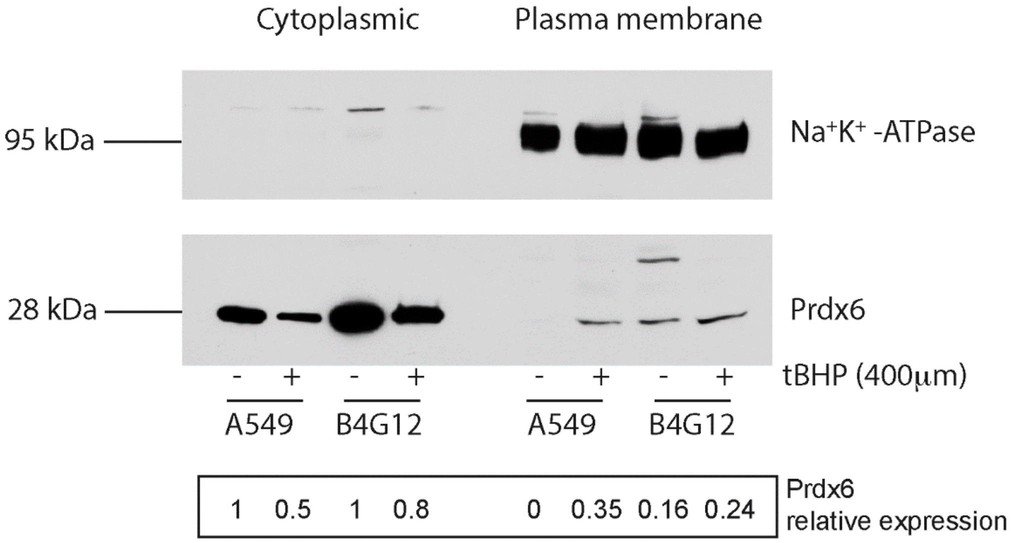

Figure S1. Cytoplasmic and plasma membrane (PM) proteins were purified from A549 and B4G12 cell lines left untreated (-) or treated (+) for 3 h with tBHP. Fractionation was performed as described in Section 2.4.1. Cytoplasmic Prdx6 levels were quantitated by densitometry relative to untreated. Plasma membrane Prdx6 was calculated by density PM/density cytoplasmic Prdx6. N.B. The second band appearing at ~50kDa in the PM fraction of untreated B4G12 cells was not consistently observed. Therefore, this is most likely to be non-specific.

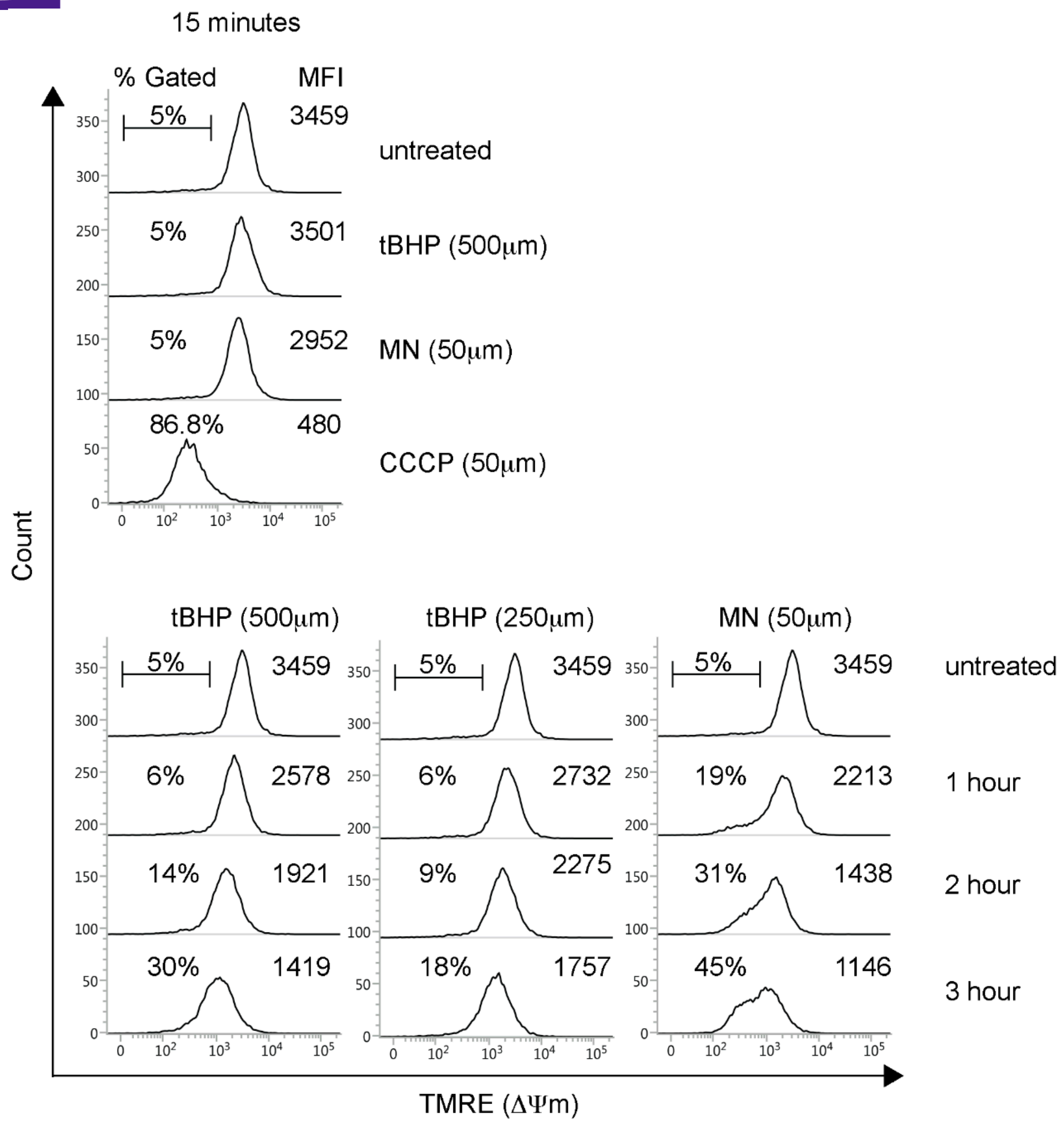

Figure S2. Menadione disrupts mitochondrial membrane potential. B4G12 cells were treated with the indicated compounds for the desired times. TMRE was added and ΔΨm measured by flow cytometry.
